# The Effect of Vegetation Ecological Restoration by Integrating Multispectral Remote Sensing and Laser Point Cloud Monitoring Technology

**DOI:** 10.3390/plants13223164

**Published:** 2024-11-11

**Authors:** Mengxi Shi, Shuhan Xing, He Bai, Dawei Xu, Lei Shi

**Affiliations:** 1 College of Landscape Architecture, Northeast Forestry University, Harbin 150040, China; 2 Key Laboratory for Garden Plant Germplasm Development & Landscape Eco-Restoration in Cold Regions of Heilongjiang Province, Harbin 150040, China; 3 College of Horticulture and Landscape Architecture, Heilongjiang Bayi Agricultural University, Daqing 163319, China

**Keywords:** MRS, laser point cloud monitoring, vegetation ecology, Daqing oilfield, coverage area

## Abstract

This research aims to evaluate and monitor the effectiveness of vegetation ecological restoration by integrating Multispectral Remote Sensing (MRS) and laser point cloud (LPC) monitoring technologies. Traditional vegetation restoration monitoring methods often face challenges of inaccurate data and insufficient coverage, and the use of MRS or LPC techniques alone has its limitations. Therefore, to more accurately monitor the vegetation restoration status, this study proposes a new monitoring method that combines the advantages of the large-scale coverage of MRS technology and the high-precision three-dimensional structural data analysis capability of LPC technology. This new method was applied in the Daqing oilfield area of China, aiming to provide effective ecological restoration assessment methods through the precise monitoring and analysis of regional vegetation growth and coverage. The results showed that there was a negative correlation between the vegetation humidity index and vegetation growth in the Daqing oilfield in 2023. The estimated monitoring effect of the research method could reach over 90%, and the coverage area of *hydrangea* restoration in the monitoring year increased by 7509 km^2^. The research technology was closer to the actual coverage situation. The simulation image showed that the vegetation coverage in the area has significantly improved after returning farmland to forests. Therefore, the technical methods used can effectively monitor the ecological restoration of vegetation, which has great research significance for both vegetation restoration and monitoring.

## 1. Introduction

With the intensification of climate change, land degradation, and biodiversity loss, ecological restoration has become an important measure for the global response to environmental challenges. Effective monitoring can provide decision support, optimize resource allocation, reduce costs, and increase the probability of successful ecological restoration. The intensification of global environmental degradation has made vegetation ecological restoration (VER) an important link in ecological environment construction. Vegetation can not only improve the ecological environment and increase biodiversity but also enhance land use efficiency and regional climate regulation capacity [1]. Therefore, it is of great significance to accurately monitor the effectiveness of vegetation restoration to evaluate the effectiveness and sustainability of restoration projects [2]. Multispectral Remote Sensing (MRS) technology can provide large-scale vegetation coverage information, ecological change data, and vegetation health status reflected by different spectral bands. Meanwhile, the unique Normalized Difference Vegetation Index (NDVI) and other parameters of MRS technology can be used to measure the density and vitality of vegetation [3]. Although MRS technology has advantages in coverage and data acquisition speed, it lacks the ability to analyze the three-dimensional (3D) structure of vegetation in detail, making it difficult to accurately capture structural data such as vegetation height and crown width. Laser point cloud (LPC) technology can provide high-precision 3D point cloud data and accurately evaluate the physical structure of vegetation. This technology is particularly suitable for scenarios that require precise 3D analysis, such as the accurate measurement of vegetation height and coverage. Although LPC technology performs well in detailed analysis, its coverage is usually limited, and the cost is high, making it unsuitable for large-scale continuous monitoring. Therefore, by combining MRS and LPC technologies, the research can simultaneously utilize the wide area coverage capability of MRS and the high-precision 3D structure analysis of LPC, thereby enhancing the monitoring and analysis capabilities of vegetation restoration effects. The Geographic Information System (GIS) can provide spatial data integration and support the dynamic monitoring and management of ecological changes. However, MRS has limitations in structural details, and LPC still has issues with poor monitoring effectiveness in wide area coverage and ecological restoration monitoring. Therefore, a new monitoring framework is proposed by combining various remote sensing and point cloud technologies to enhance the monitoring capability of ecological restoration processes.

Numerous industry scholars have achieved significant research results in monitoring ecological restoration. Camarretta N et al. found that there are still some issues in evaluating the success of ecological restoration and, therefore, proposed a near-end sensing technology [4]. This technology used handheld laser scanners to measure the annual changes in the horizontal features of trees and also utilized dense point clouds to summarize various structural features of trees. The characteristics derived from this method are increasing year by year. It had high accuracy in monitoring the temporal changes in forest features, providing a reference for evaluating recovery trajectories. Nuijten R J et al. found that ordinary vegetation measurement methods have higher costs and, therefore, proposed an unmanned aerial vehicle digital aerial photogrammetry method [5]. This method evaluated the spatial arrangement and DPA height of vegetation through cluster analysis using land cover maps, distribution spatial relationships, and patch sizes. This method could determine the implementation sequence of future inspections and mitigation measures and quickly identify the number and types of vegetation in the area. Tian J et al. found that field measurements of ecological restoration are very time-consuming and labor-intensive and, therefore, proposed a new method for measuring vegetation structure parameters [6]. This method quantitatively studied individuals and used laser scanning technology to quantify vegetation coverage and leaf area index. The experiment showed that the vegetation parameters measured by this method were basically consistent with the on-site measured data, and the recognition rate of shrubs improved by 60%. Ventura et al. found that seagrass ecosystems are facing significant threats and, therefore, proposed a restoration area detection method based on underwater SFM [7]. This method used RGB dense images, 3D dense point clouds, and grating layers to characterize the seabed and combined image classification techniques. This method could effectively monitor changes in seagrass ecology and provide effective support for the protection of underwater ecosystems. Using a meta-analysis of multiple articles, He T et al. found that studies show that current forms of forest and grassland restoration increase methane uptake by 90.0% and 30.8%, respectively [8]. It is evident that the significant effect of greenhouse gas emissions can be reduced through vegetation restoration, but how to monitor the effect of vegetation restoration needs to be further explored. In the study by Li C et al., it was found that vegetation restoration enhances soil properties, and for the study data, observations, and data analysis were carried out in different studies. The results of the study showed that vegetation restoration can play a key role in post-mining land rehabilitation [9]. Although the study analyzed the soil condition with vegetation restoration, the observation of the effect of vegetation restoration in the current study needs to be further explored. Thakur TK et al. proposed the use of geospatial techniques and ground-based measurements in order to study land cover changes and spatial and temporal variations in forest carbon stocks and soil organic carbon in central India. The results of the study showed significant changes in vegetation carbon stock and soil organic carbon content as a result of the reduction in forest area and expansion of agricultural and residential areas [10]. This study investigated the changes in vegetation restoration and soil organic carbon content, but further investigation and analysis are needed to monitor the effects of vegetation restoration. Yang Y et al. proposed a method using laser imaging, detection and ranging (LiDAR), and hyperspectral data in order to study the important role of different elements in ecological restoration and their distributional characteristics. The results of the study showed that by extracting spectral, textural, and height characteristics of vegetation as well as vegetation indices and structural parameters, effective models can be developed to predict the distribution of C, N, and P [11]. This study investigated the content situation of different elements, but the restoration effect and restoration situation of the indexes need to be further explored. In order to explore the application of remote sensing technology in ecological restoration monitoring, Wang R et al. analyzed data from the literature in the field of ecological remote sensing, and the results of the study showed that remote sensing technology plays a key role in ecological restoration monitoring [12]. Although remote sensing technology can be used to monitor ecological restoration, the actual vegetation cover and growth still need to be further explored.

In summary, traditional research methods lack sufficient monitoring of vegetation restoration and the problem of inaccurate information expression also brings new challenges to the monitoring of VER effectiveness. Therefore, this study innovatively proposes a technology combining MRS imaging and LPC to improve the ecological monitoring effect under different conditions. Meanwhile, this study also analyzes the growth correlation of vegetation to calculate the optimal growth area and environment for vegetation. By combining the advantages of these two technologies, it is possible to comprehensively analyze the growth status, biomass, and ecological function of vegetation, thereby more accurately evaluating the effectiveness of VER. Combining the advantages of different technologies can not only overcome the problem of the poor monitoring effectiveness of VER but also greatly reduce monitoring costs, thereby enhancing the data analysis and monitoring capabilities of vegetation ecosystems. The goal of this research is to provide a comprehensive method that can monitor vegetation restoration with high precision and efficiency on a large scale, thereby providing solid data support and a decision-making basis for ecological restoration projects.

## 2. Materials and Methods

### 2.1. Basic Overview of Vegetation Areas

Daqing oilfield is one of the famous oil and gas fields in China. As the largest oil production base in China, the Daqing oilfield has produced more than 2.4 billion tons of crude oil over the past 60 years of continuous crude oil extraction, making great contributions to China’s economic construction. With the increasing amount of oil extraction, the area of oilfield extraction has also been expanding, and as of 2022, the area of oilfield extraction, i.e., the area of environmental disturbance—has reached 1399.2 km^2^. During the oil extraction process, highly dispersed oil pollutants from point sources inevitably enter the environment. Due to insufficient environmental protection concepts and negligence in prevention and control, the pre-industrial development of the Daqing oilfields has neglected environmental protection and caused some damage to it, with the most serious damage caused to the vegetation in the oilfield production area [13,14].

The Daqing oilfield area is located in the western part of Heilongjiang Province, China, and belongs to the southern part of the Songnen Plain, where the crustal structure is relatively stable. Due to numerous tectonic activities in its geological history, a series of faults and fissures have been formed around the Daqing oilfield, and these tectonic features have had a significant impact on the topography and distribution of resources in the region. The main geomorphology of the oilfield area is dominated by plains with flat terrain, which is suitable for large-scale oil extraction activities. Vegetation types in the region are diversified according to the influence of climate zones and human activities. In the lower altitude area of the plain, meadow grassland and farmland are mainly distributed, in which common plants include drought-tolerant grass species such as sheep grass and *brown cryptomeria*, as well as large areas of rice, corn, and soybean cultivation. With the increase in human activities, the original vegetation has been replaced by large-scale agricultural land. Since 2000, the Daqing region has been focusing on ecological protection and restoration along with oil extraction and urban expansion. The implementation of policies aimed at returning farmland to forests (RFTF) and the establishment of reforestation programs has facilitated the planting of a significant number of tree species that are well-suited to the local climate. These include species such as Birch and camphor pine, which have been planted with the objective of restoring and increasing the green coverage of the region. Furthermore, the region is engaged in the implementation of soil and water conservation programs, as well as the establishment of an ecological monitoring network. This network is designed to observe and assess changes in vegetation, biodiversity, and ecosystem health, aiming to minimize the environmental impact of oil extraction activities. On the one hand, the development of the Daqing oilfields requires the removal of a certain amount of natural vegetation for the construction of the extraction facilities, which causes some disturbance and damage to the natural environment. On the other hand, ecological restoration and revegetation of the areas surrounding the oilfields have also become an important part of environmental protection. Therefore, Daqing oilfield has taken a series of environmental protection measures during the development process, such as afforestation, wetland protection, and restoration, to mitigate the impact of development activities on the environment.

### 2.2. Monitoring and Analysis of Vegetation Restoration Data

**Figure 1:**
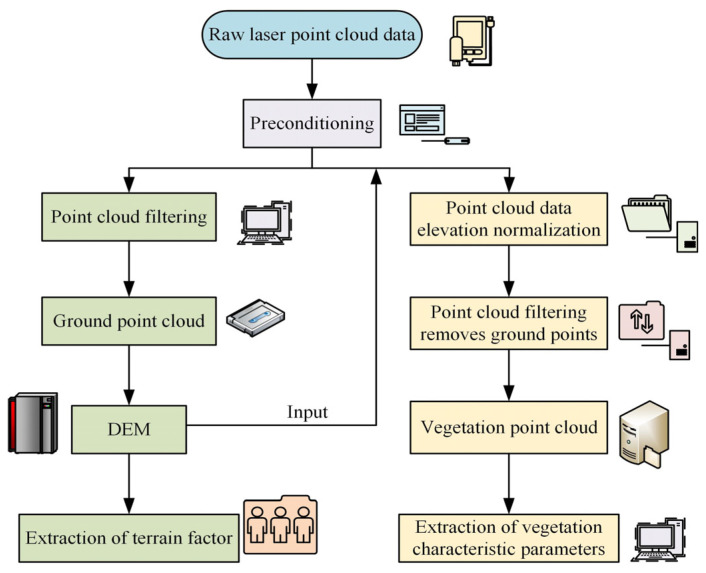
Data processing flow of LPC monitoring technology.

To monitor the effect of vegetation restoration in the Daqing oilfield area in real-time, drone MRS technology is used to monitor the vegetation growth, vegetation distribution, and other data in the Daqing oilfield area. Furthermore, characteristic data on the distribution of vegetation growth characteristics in this region have been obtained [15]. This study uses LPC monitoring technology to analyze vegetation distribution data in the region. Figure 1 shows the data processing flow of LPC monitoring technology.

In Figure 1, after obtaining the initial vegetation indicator data, the data are preprocessed and analyzed. The preprocessing process can be divided into two steps: point cloud filtering and data normalization. The former generates point cloud research areas from the obtained vegetation data and analyzes the data factors of the extracted areas through point cloud research. The regional data factors are normalized, and the ground data points are removed and filtered by the point cloud to obtain the data point cloud of vegetation distribution. Finally, vegetation feature data parameters are extracted. The preprocessing data process can also be directly normalized, simplifying the process of obtaining vegetation point clouds.

**Figure 2:**
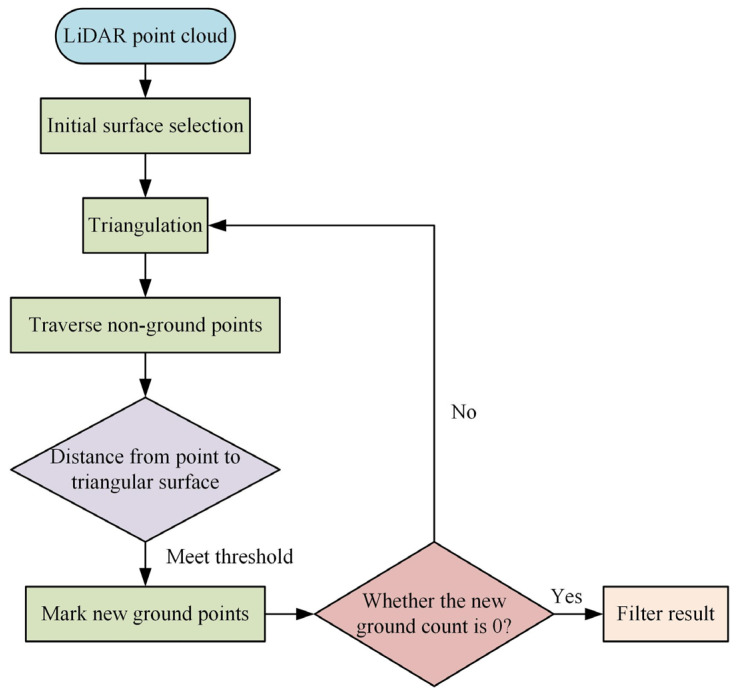
Algorithm flowchart.

To obtain a more precise point cloud research area model, it is necessary to filter and process the obtained data point clouds, excluding point clouds other than the vegetation point clouds obtained through drone MRS. The Daqing oilfield area is characterized by diverse grassland and wetland vegetation. To obtain a more high-definition vegetation point cloud data set, our research project employed a progressive encryption triangular mesh filtering method for data filtering processing [16]. The algorithm process is shown in Figure 2.

In Figure 2, the PETF method requires first filtering the data point cloud to obtain the initial ground point positions. The length of the building is used as the length of the data border to digitize all point cloud grids. The initial point cloud data in the grid are extracted as seed point cloud data. Based on the initial point cloud data, a point cloud triangulation network was constructed to query all point cloud data. Whether the distance angle of triangulation meets the threshold setting is judged. If it meets the requirements, a new location cloud is marked. If it does not meet the requirements, the location cloud will be filtered again. The final step is to determine if the new ground point cloud is 0. If so, the filtering result is output. If not, a new triangulation is established. Vegetation numerical analysis uses normalization methods to analyze vegetation point clouds and calculates and analyzes data using infrared bands in MRS images. The vegetation index data for calculating point clouds is given by Equation (1), which is the formula for calculating data bands [17].

(1) $$NDVI=\frac{NIR-R}{NIR+R}$$

In Equation (1), NDVI represents the band. NIR represents the near-infrared band of the MRS image. R represents the red-light band. Due to the deviation in the calculation of different MRS images, the calculation in different MRS images is given by Equation (2).

(2) $$NDVI_{L5}=\frac{L5_{-}B4-L5_{-}B3}{L5_{-}B4+L5_{-}B3}$$

In Equation (2), $NDVI_{L5}$ represents vegetation image data in band L5. $L5_{-}B4$ represents the near-infrared band at L5, and $L5_{-}B3$ represents the red band at L5. The degree of vegetation coverage is an important indicator for measuring the vegetation situation in a region. The degree of vegetation coverage is determined by the ratio of the projected area of the vegetation species structure to the total ground area. Equation (3) is the vegetation coverage formula [18].

(3) $$FVC=\frac{NDVI-NDVI_{moveg}}{NDVI_{allveg}-NDVI_{moveg}}$$

In Equation (3), FVC represents vegetation coverage. $NDVI_{moveg}$ and $NDVI_{allveg}$ represent the MRS image bands of non-vegetated areas and vegetated areas in the current region. Due to the fact that vegetation coverage is often influenced by time, the Sen Slope Estimation Method (SenSEM) is often used to calculate the stability of parameters and noise interference in the analysis of vegetation coverage. Equation (4) is the vegetation coverage test formula [19].

(4) $$k=median[(f_{j}-f_{i})/(j-i)],\forall j>i$$

**Figure 3:**
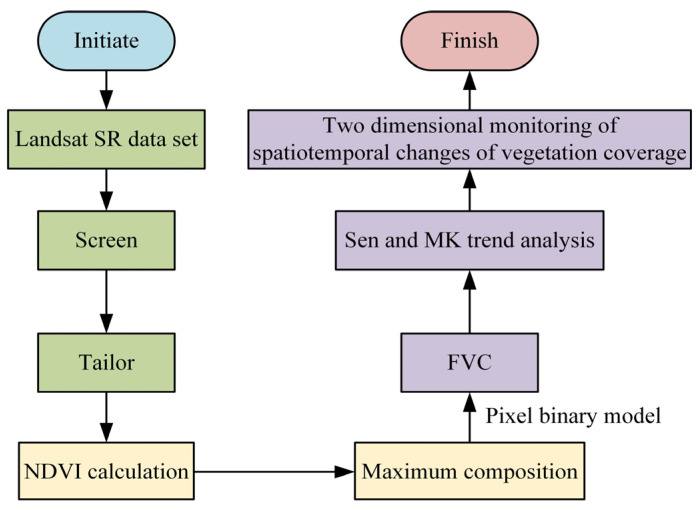
Vegetation plane monitoring process.

In Equation (4), k represents the median vegetation coverage. $f_{j}$ and $f_{i}$ are the vegetation coverage of the MRS image elements in years j and i. The median represents SenSEM. SenSEM is used to analyze the changes in vegetation coverage in the region in the past. To accurately predict and analyze the vegetation coverage and growth situation, planar monitoring methods are used to monitor and analyze the vegetation restoration situation. Figure 3 shows the process of vegetation plane monitoring.

In Figure 3, when monitoring vegetation conditions, it is necessary to analyze the distribution and growth of vegetation through past vegetation databases, including screenings and crops, and eliminate vegetation data information with a large time span. The NDVI calculation method is adopted to calculate the NDVI value of vegetation, and then the maximum NDVI value of the calculation result is taken as the maximum value of the current period. The vegetation FVC value is calculated through the NDVI value, and the current coverage and growth trend of vegetation based on SenSEM are analyzed. Finally, fixed time and spatial vegetation growth monitoring data were obtained.

### 2.3. Analysis of Ecological Effects of Vegetation Restoration

To better analyze the vegetation ecological effects generated by LPC and MRS technologies, this study uses the Digital Elevation Model (DEM) for analysis. In the process of building DEM, a two-dimensional grid plane structure is constructed in the point cloud space, and different point cloud grids are defined in scale to calculate the elevation value of the grid. The data sampling points in the DEM are obtained through interpolation, thereby obtaining the DEM of vegetation cover data. To accurately predict the grid situation of power supply data, this study uses an irregular triangular interpolation method to calculate grid interpolation. This method involves two modeling processes, one of which is to build a DEM for the region based on the initial sampling points and perform interpolation calculations on the model. Equation (5) represents the interpolation calculation process [20].

(5) $$Q_{u}=\{\begin{array}{c} \frac{1}{D_{u}},\;D_{i}>0,and,D_{i}\leq Width\\ \frac{1}{D_{u}^{2}},\;0<D_{i}\leq \frac{Width}{2}\\ {\left(\frac{Width}{D_{u}}\right)}^{2},\frac{Width}{2}<D_{i}<Width \end{array}$$

In Equation (5), $Q_{u}$ represents the weight value of the elevation model. $D_{u}$ represents the distance between the network insertion methods. Width represents the search window size for network interpolation. The elevation value of the model is calculated as shown in Equation (6).

(6) $$E=\frac{\sum_{i=1}^{Nc}Q_{u}E_{u}}{\sum_{i=1}^{Nc}Q_{u}}$$

**Figure 4:**
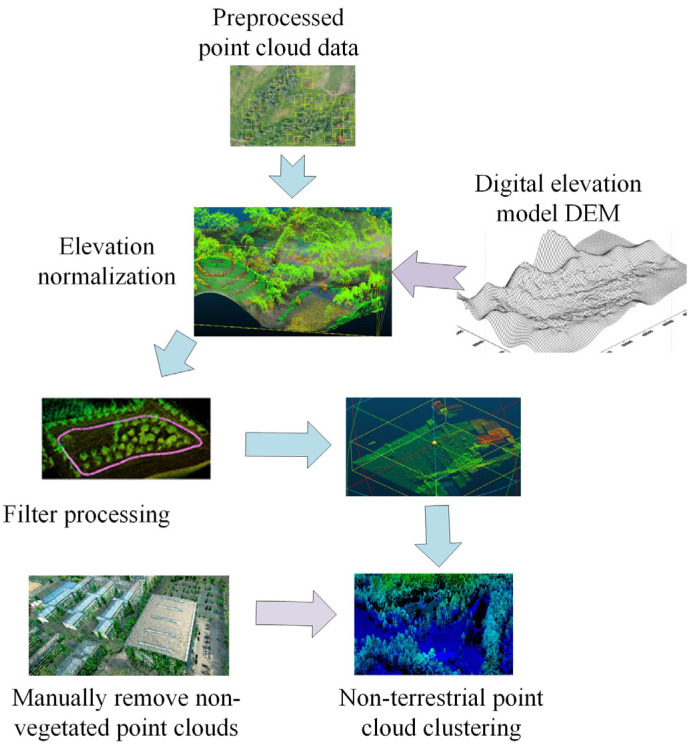
Vegetation data extraction process.

In Equation (6), E represents the elevation value size of the model. $E_{u}$ represents the elevation value of the model at point u. Nc represents the number of sampling points in the window of the model. The geomorphology of the selected study area, comprising wetland and grassland landscapes, has resulted in the dominance of herbaceous plants across most vegetation types. This has led to a wide distribution of vegetation and overlapping canopies, which has rendered the data obtained from remote sensing technology redundant and repetitive. Consequently, a more fine-grained extraction of the data is required. Figure 4 shows the process of extracting vegetation data.

In Figure 4, during the processing of point cloud data, it is necessary to preprocess the point cloud data first. DEM normalizes point cloud data, filters point cloud data, and clusters non-vegetation point cloud data. At the same time, redundant point cloud data are removed and subjected to conditional constraints to output the obtained point cloud data. The normalization process is to eliminate the impact of regional mountain fluctuations on point cloud data. The calculation of the slope is an important indicator for analyzing the degree of steep slope inclination in a model. Different degrees of undulation result in different slopes and terrains, leading to more climate and soil erosion in different situations, which also have varying degrees of impact on the ecological restoration of vegetation. Equation (7) provides the slope calculation formula [21].

(7) $$Slope=ar\cos(\frac{z-f}{\left|z\right|\times \left|f\right|})$$

In Equation (7), Slope represents the slope. z represents the vertical coordinate value of the model. f represents the normal vector of the model, and another way is shown in Equation (8).

(8) $$Slope=ar\tan(\sqrt{H_{x}^{2}+H_{y}^{2}})\times 180/\pi$$

In Equation (8), $H_{x}$ and $H_{y}$ represent the horizontal and vertical increments of the elevation model on the x and y axes. By calculating and analyzing the slope values, the slope changes in the model can be accurately analyzed to assess the vegetation situation in the area. The direction of the slope is an important indicator for measuring slope conditions. The distribution of the slope direction will affect the growth of vegetation on different slopes. Vegetation with different slope orientations receives different levels of solar radiation and light exposure, resulting in significantly different growth conditions. Equation (9) is the formula for the slope direction value [22].

(9) $$Aspect=ar\tan(\frac{H_{y}}{H_{x}})$$

In Equation (9), Aspect represents the magnitude of the slope direction value. The distribution of humidity in a region is an important factor in determining the rainfall situation in the region. The larger the humidity index, the better the precipitation and water-locking ability of the region. The smaller the relative humidity index, the worse the water-locking ability of the region with smaller precipitation. Equation (10) is the humidity index formula.

(10) $$TWI=\ln(SHCA/\tan\theta_{-}scale)$$

In Equation (10), TWI is the size of the humidity indicator value. $\theta_{-}scale$ is the unit calculation result value of the grid slope corresponding to the humidity indicator. SHCA is the unit catchment area of the region. The formula for the catchment area is shown in Equation (11) [23].

(11) $$SHCA=HCA/Flowfinaldir_{-}length$$

In Equation (11), $Flowfinaldir_{-}length$ is the size of the catchment width in the region. HCA represents the catchment area of each unit. The formula for HCA is given by Equation (12) [24].

(12) $$HCA=Flow_{-}Acc\times cell_{witch}\times cell_{widch}$$

**Figure 5:**
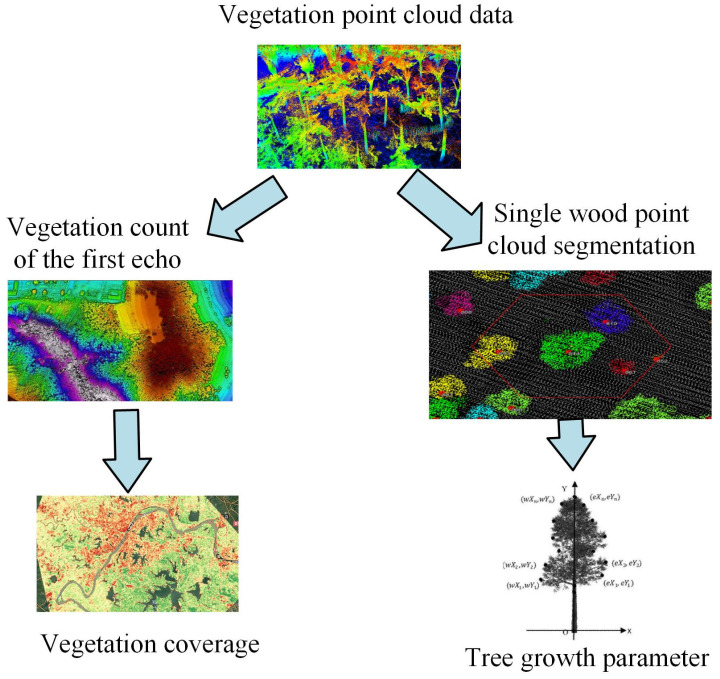
Extraction process of vegetation growth data.

In Equation (12), $Flow_{-}Acc$ represents the catchment area matrix. $cell_{witch}\times cell_{widch}$ represents the product of the unit catchment area. Data information on the growth of different types of vegetation, such as the number of herbaceous plants, trees, vegetation height, vegetation restoration, and vegetation restoration status, needs to be analyzed through growth parameters. Figure 5 shows the process of extracting vegetation growth data.

In Figure 5, the extraction process of vegetation parameters is divided into two directions. One is to count the number of vegetation point clouds that have appeared for the first time in the current collection of MRS technology and point cloud data and calculate the vegetation coverage degree of vegetation through data parameters. On the other hand, the growth of trees is calculated based on parameters such as the amount of vegetation, height, number, and the crown width of a single tree. The degree of vegetation cover is the percentage of tree vegetation cover repetition in some areas with a tree canopy in relation to the total area of vegetation, which is significantly different from the coverage rate of vegetation. The formula for vegetation coverage is given by Equation (13).

(13) $$VC=\frac{N_{vegfirst}}{N_{first}}$$

**Figure 6:**
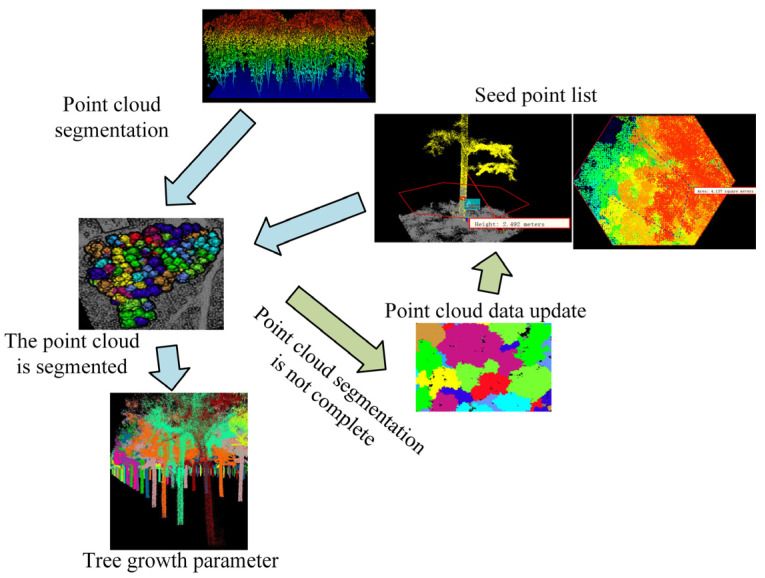
Data acquisition process.

In Equation (13), VC represents the degree of vegetation coverage. $N_{vegfirst}$ represents the number of vegetation point clouds covered when the vegetation height exceeds the specified height for the first time. $N_{first}$ represents the total number of point clouds when the vegetation height exceeds the specified height for the first time. The acquisition of new parameters based on vegetation growth in the Daqing oilfield area, along with the data acquisition process, is illustrated in Figure 6.

In Figure 6, after obtaining vegetation point cloud data, the data parameter process needs to first segment the vegetation point cloud parameters to determine whether the current point cloud has been fully segmented. If all are completed, the growth parameter data information of the current vegetation will be output. If it is not completed, when updating the vegetation point cloud data, the current state of the vegetation seed point cloud parameter data is listed first, and then the seed point cloud data are segmented. The above steps are repeated until the growth parameter data of the trees is obtained. Comparative analysis was conducted on the correlation coefficients of vegetation restoration in the study, as shown in Equation (14) [25].

(14) $$r=\frac{\sum xy-\frac{\sum x\sum y}{N}}{\sqrt{\sum x^{2}-\frac{{\left(\sum x\right)}^{2}}{N}(\sum y^{2}-\frac{{\left(\sum y\right)}^{2}}{N})}}$$

In Equation (14), r represents the magnitude of the correlation coefficient. A coefficient greater than 0 indicates a positive correlation with the variable, while a coefficient less than 0 indicates a negative correlation with the variable.

### 2.4. Experimental Data and Materials

The drone used for MRS data acquisition in this study is the DJI Matrice 300 RTK (DJI, Shenzen, China). Typical multispectral sensors include MicaSense RedE (AgEagle, Wichita, KS, USA). In the study, flight planning software such as DJI Terra 4.2.5 was used for mission planning. Firstly, the flight altitude was set to 60–120 m, and the overlap rate was set to a forward overlap rate of 75–80% and a lateral overlap rate of 60–70%. The drone flies along a preset path while continuously collecting data through multispectral sensors. Sensors capture multiple images per second. The collected image data will be downloaded in real-time or after the flight to the ground stations or cloud storage devices. The data storage format is GeoTIFF, with image location information attached. The study uses Pix4DMap 4.5.6 software to stitch and orthorectify the collected multispectral images, generating orthorectified images covering the entire target area. Specialized software or ENVI 5.6.3 is used to perform multispectral analysis on the stitched images, calculate NDVI or other relevant vegetation indices, and evaluate vegetation health status. To ensure the accuracy of the data, on-site verification is also required. When using LPC technology, the LiDAR sensor model Velodyne HDL-32ELiDAR (Mapix Technologies Ltd., Edinburgh, UK) is used. Before the data acquisition, the LiDAR sensor is carried by a drone and flown along a predetermined route. The sensor emits a laser beam and receives echoes reflected from the ground or objects. After data collection, specialized software such as RIEGL 1.9.4 RiPROCESS is used to perform preliminary processing on the original point cloud, including noise filtering, ground point classification, and the removal of unnecessary non-vegetation points. Then, the point cloud is classified using automatic or semi-automatic methods. Finally, further 3D modeling is performed on the classified point clouds to extract the required structural features and generate DEM, Digital Terrain Models (DTMs), and Canopy Height Models (CHMs).

Data and information on vegetation restoration, vegetation growth, and slope humidity in the Daqing oilfield area are monitored and analyzed to assess the effect of VER in the area. The research spans from 1990 to 2023. The 1990 time point is when the government began to emphasize the ecological restoration of vegetation in the Daqing oilfield area. The results of the correlation coefficients shown in Table 1 were obtained by analyzing the growth of vegetation in the Daqing oilfield area in this time period. Elevation is the altitude, slope is the vegetation slope, aspect is the direction of the vegetation slope, and TWI is the vegetation humidity index. Vegetation coverage (FVC) refers to the proportion of ground covered by vegetation when viewed from above and is an important indicator for evaluating vegetation coverage and ecological restoration. The range of FVC values usually varies between absolute values of 0 and 1, where an absolute value close to 0 indicates no vegetation cover and an absolute value close to 1 indicates complete vegetation cover. In the vegetation monitoring of the Daqing oilfield area, a 1 m resolution was used to generate 10,000,000-pixel points on a 10 km^2^ area. The pixels use a resolution of 0.3 m to 1 m, with 1 m representing a ground area of 1 m^2^ per pixel.

## 3. Results

### 3.1. Analysis of Regional Vegetation Ecological Situation

The spectral errors (FVC and NDVI) in this study were caused by atmospheric interference and sensor calibration, so the error range was controlled between ±5% and ±10%. The point cloud density error is allowed to be within ±10% in current research by increasing the point cloud density and improving data processing algorithms. In this study, the model parameter error was controlled within a range of ±10%. The slope and aspect errors in the study were controlled within an angle error range of ±2°. The positioning error of the final model was controlled within ±1 pixel. All the data information and error control ranges used in the study are within the limited error range. This study improves the accuracy of model analysis by excluding data with larger errors.

**Figure 7:**
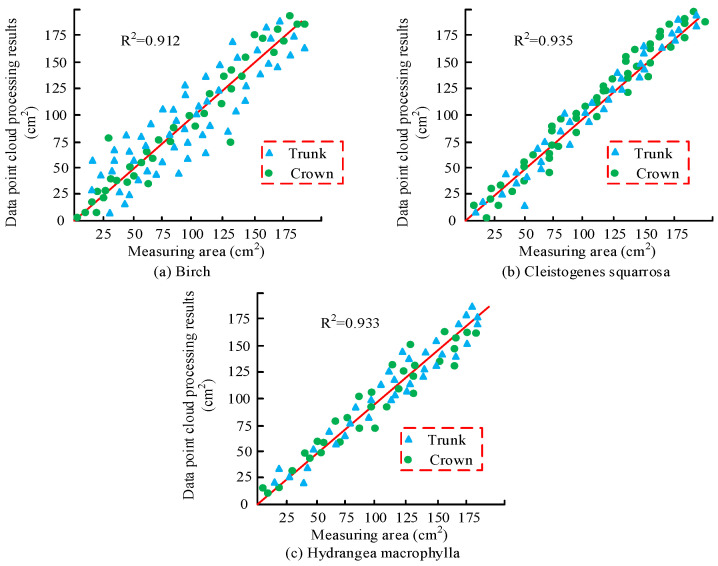
Monitoring the growth of different plants.

In Table 1, during 2023, the vegetation humidity index in the Daqing oilfield area showed a negative correlation, which means that the worse the vegetation growth, the lower the vegetation humidity index. During 2023, other indicators of vegetation showed a positive correlation, indicating that higher values of altitude, slope, and vegetation slope direction lead to better growth of vegetation. Between 2023 and 2010, the growth status of vegetation was positively correlated, indicating that the better the growth status of vegetation, the higher the indicator value of vegetation. The changes in several indicators over the past 33 years show that the correlation between vegetation growth indicators in this region has shown a positive growth trend since 1990, which may be due to the increasing effect of VER year by year. To test the growth of vegetation under different growth conditions, the growth of the three plants, *white Birch*, *brown cryptomeria*, and *hydrangea*, was measured and compared, as shown in Figure 7. The R^2^ value represents the accuracy between the monitoring situation and the actual measurement situation. The data in the figure represent the actual deviation between the monitored values and the true values. When the true values are the same as the monitored values, the deviation in the R^2^ value in the figure is one. The larger the deviation between the monitored value and the true value, the closer the deviation of the R^2^ value is to 0.

**Figure 8:**
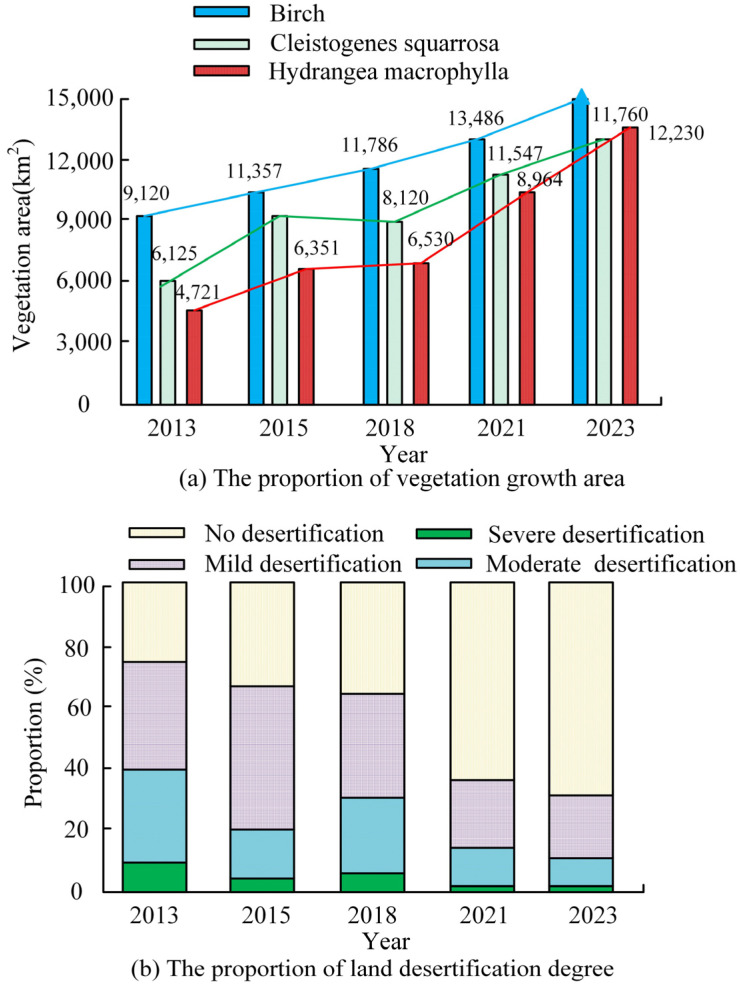
Analysis of vegetation growth and ecological restoration.

In Figure 7a, in the comparison between the data point cloud and the actual situation, the monitoring effect of Birch’s actual measurement and data point cloud monitoring can reach 91.2%. Among them, the change in the crown of the Birch tree is closest to the true value, while the deviation in the trunk part is relatively large, which may be due to the dense branches of the Birch tree obstructing the observation of the trunk. In Figure 7b, the monitoring effect of the growth of *Cryptotaenia scabra* can reach 93.5%, which may be due to the dense point cloud data of *Cryptotaenia scabra*. In Figure 7c, the monitoring effect of *hydrangea* growth can reach 93.3%, which may be due to the easier observation of *hydrangea* growth. The three graphs indicate that the monitoring effect of vegetation with dense branches is relatively low, but the overall monitoring effect can also reach over 90%, indicating that the integration of point cloud and MRS technology can effectively monitor the ecological situation of vegetation. Analyzing the past ten years, the degree of vegetation restoration in the Daqing oilfield area is comparable, as shown in Figure 8.

In Figure 8a, the vegetation growth of different plants shows an increasing trend year by year over the past decade. The changes in Birch over the past decade have increased from the coverage proportion of 9120 km^2^ in 2013 to 14,660 km^2^ and the coverage area has increased by 5540 km^2^. This indicates that the vegetation restoration of Birch has significantly improved in the past decade. The growth coverage of *Cryptotaenia scabra* and *hydrangea* has increased by 5635 km^2^ and 7509 km^2^, respectively, in the past decade. The vegetation growth in the Daqing oilfield area is clear, which may be the reason why RFTF has been vigorously implemented in recent years. In Figure 8b, after the vegetation growth improved, the degree of land desertification in the Daqing oilfield also improved. Up to now, more than 60% of the regions have not experienced land desertification, which is due to the result of vegetation protection.

### 3.2. Analysis of Vegetation Restoration Monitoring Effectiveness

**Figure 9:**
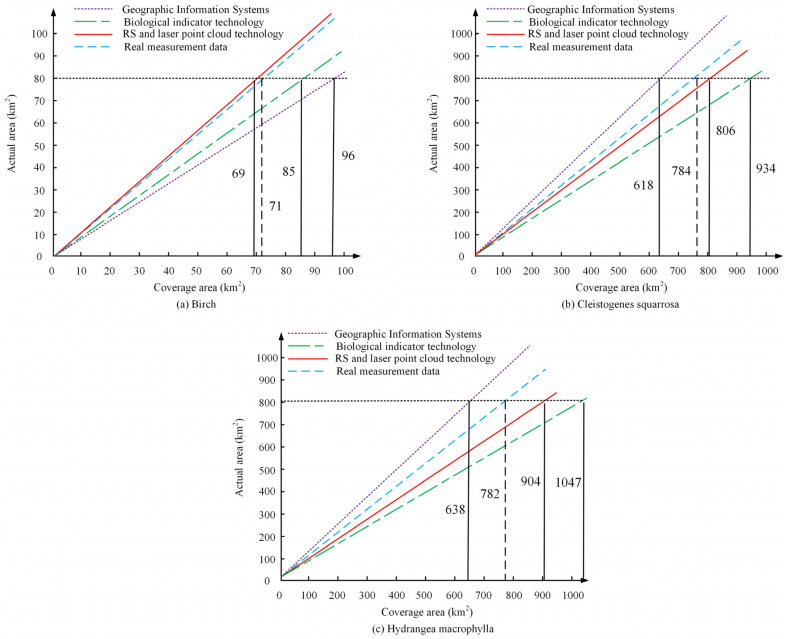
Comparative effects of different technologies on three types of plants.

To test the effect of vegetation monitoring in the Daqing oilfield area under different technological conditions, the technology used in this study was compared with GIS and Biological Indicator Technology (BIT) for vegetation coverage area monitoring. The results of comparing the vegetation coverage of three plants, namely Birch, red leaf rhododendron, and *hydrangea*, are shown in Figure 9. GIS is a framework for collecting, managing, and analyzing spatial and geographic data. In ecological restoration, GIS can be used to draw vegetation cover maps and monitor changes over time. BIT is used to evaluate environmental conditions. BIT is commonly used for vegetation monitoring, which helps determine the effectiveness of restoration by measuring changes in the abundance, distribution, and health status of indicator species.

In Figure 9a, the real measurement of 80 km^2^ is used as an example in the monitoring of Birch’s coverage. The area covered by *white Birch* in the method used in this study was able to reach 69 km^2^, which is closer to the true coverage of 71 km^2^ compared to 96 km^2^ in the GIS. From Figure 9b, the cover area of *brown cryptomeria* is closer to that of the real situation with the real measurement of 800 km^2^; for example, the cover area is able to reach 806 km^2^, which is 22 km^2^ different from the real cover area. In Figure 9c, taking a real measurement of 800 km^2^ as an example, there is a significant deviation between the research method and the actual coverage area, with an area difference of up to 122 km^2^. This may be due to the measurement bias caused by the characteristics of *hydrangea* plants. The integration of MRS and LPC technology can effectively monitor the ecological status of vegetation. Table 2 shows the vegetation analysis of different regions.

**Figure 10:**
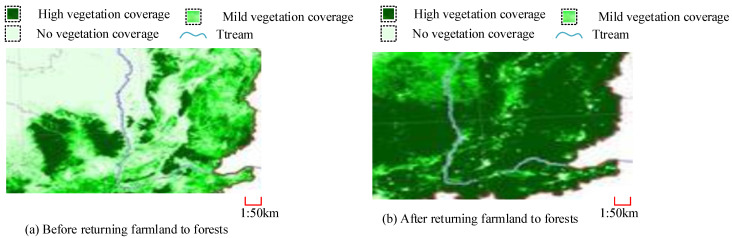
Vegetation coverage before and after RFTF in Mount Tai.

In Table 2, there are differences in the growth and coverage of vegetation in different regions, which may be due to different soil and water conditions in the region. The area with the highest number of single trees is area 3. For example, the maximum height of vegetation in area 3 can reach 13.64 m, and the maximum width of vegetation can reach 7.68 m. In terms of coverage in this area, vegetation coverage can reach 73.68%. Due to the complexity of the situation between regions, vegetation with a height of less than 0.1 m in point cloud data in different regions was not included, resulting in small errors between the data. However, from the overall data, the number of individual trees could not accurately reflect the growth of vegetation, while the height and width of vegetation did not accurately reflect the growth of vegetation, some of which was not accounted for. Figure 10 shows a comparative analysis of vegetation coverage using LPC and MRS technology.

Figure 10a,b show that the vegetation coverage and vegetation depth in the Daqing oilfield area significantly improved before and after RFTF. The darker the color, the denser the vegetation in the area. After RFTF, there was a significant increase in vegetation density, depth, and coverage area. This indicates that RFTF can significantly improve VER performance, and the use of MRS and LPC can also achieve the monitoring of VER conditions.

## 4. Discussion

In this manuscript, a real-time monitoring model for ecological restoration in the Daqing oilfield area using MRS and LPC technology is proposed. The use of MRS and LPC technologies can enhance the monitoring of ecological restoration effects in the Daqing oilfield area. This provides stronger data support for ecological restoration and monitoring in this region. Some comparisons to previous studies have been made. In Liu X et al.’s study, a comprehensive new method for assessing ecosystem status was provided by combining multiple vegetation indices and satellite observation techniques [26]. This method could handle complex nonlinear relationships, improving the accuracy and reliability of analysis. However, the new method relied on the quality and completeness of the input data, and any deviation or error in the input data would directly affect the accuracy of the analysis results. The new method had poor effectiveness due to the incomplete consideration of other ecological factors such as soil and biodiversity. In Tiantian C et al.’s study, the use of NDVI data from a long time series to analyze the trend of changes in vegetation resilience could reveal long-term ecological restoration effects [27]. This study quantified vegetation resilience and its nonlinear trends under different environmental gradients. However, autocorrelation analysis was mainly applicable to time series data but might overlook the impact of complex spatial heterogeneity and nonlinear factors on vegetation resilience. There are still shortcomings in the discussion of vegetation type diversity in research. This research aimed to analyze and study different vegetation types in Daqing oilfield through various techniques to achieve the goal of ecological restoration monitoring. In Ma S et al.’s study, comprehensive evaluation and elasticity analysis methods were used to determine the response thresholds of ecosystem services to vegetation cover under different vegetation types and altitude gradients [28]. This research could reveal the relationship between ecosystem service and vegetation changes. However, there were certain shortcomings in the research on refining diversity and long-term dynamic changes in ecosystem service indicators. In summary, in previous studies on the effectiveness of ecological restoration, there have been many methods that can monitor the ecological restoration effect. However, there are still many shortcomings in the current methods for monitoring the effectiveness and vegetation type. Therefore, the study in this manuscript achieved the research and monitoring of the effectiveness of new ecological restoration through the combination of multiple technologies. In addition, the study by Yu Y et al. quantified the vegetation dynamics and their relationship with precipitation in various reaches of the Yellow River Basin using satellite data on the leaf area index, net primary productivity, and precipitation use efficiency (RUE) [29]. The results of the study showed that there were significant differences in the distribution and trends of precipitation use efficiency in the upper, middle, and lower reaches. Although the study revealed a complex relationship between vegetation dynamics and precipitation, it did not fully explore the relationship between vegetation greening and enhanced ecosystem functioning. The study also ignored other key environmental variables such as temperature and land use change. The study by Mao Z et al. evaluated soil properties using artificial soil spraying as an environmentally friendly method for restoring bare slopes [30]. The experimental results showed that the combination of cement and soil aggregate has potential applications for the ecological restoration of vegetation on steep slopes. The study provides valuable guidance for practical environmental restoration. However, the study needs to be further verified as to how variables such as the actual results under field conditions and climate change affect restoration. In a study by Mazlan SM et al., the effects of forest landscape restoration were monitored using light detection and ranging remote sensing techniques, and the results of the study showed that this method was able to monitor the canopy of trees with good results [31]. The study was able to realize the monitoring of trees, but the monitoring effect on other vegetation types, such as shrubs, needs to be further explored. In the study by Santos EE et al., laser scanning technology was used to evaluate and study vegetation restoration after river accretion, and this technology was effective in analyzing surface soil deposition and monitoring restoration [32]. The study was able to monitor and analyze sediment in the river, but further research is needed to monitor vegetation restoration. In the study by Yu Y et al., the operation and maintenance of power systems were monitored using the laser point cloud technique and Euclidean clustering. The results of the study showed that the method of using laser point cloud technology and Euclidean clustering has a better monitoring effect on power system lines [33]. It can be seen that laser point cloud technology can effectively monitor the situation of different neighborhoods, but whether the method can be applied to the monitoring of vegetation restoration still needs further research. A literature analysis of the applications of laser point cloud data was carried out by Abreu N et al. It was found that laser point cloud technology can be applied to indoor–outdoor reconstructions and data monitoring [34]. Although laser point clouds were found capable of use for data monitoring in a number of areas, their analysis for the monitoring of vegetation restoration is the focus of the current research. The restoration of vegetation and ecology was monitored using UAV technology in the study by Zhang Y et al. The study showed that the use of UAV technology was able to monitor and analyze the tree height of vegetation and the carbon storage of vegetation [35]. Although the study was able to monitor vegetation restoration, the monitoring of vegetation using drone technology was poor, so the study needed to improve monitoring. Compared with the studies of other scholars, this study in this manuscript can better balance accuracy and wide-area coverage. At the same time, this research uses MRS technology to obtain a large range of vegetation health status data and combines LPC technology to make up for the shortcomings of a single technology, effectively improving the accuracy of vegetation restoration effect monitoring.

For the comparison of regional vegetation correlation, the higher the altitude, vegetation slope, and vegetation slope direction values, the better the growth status of vegetation, and the better the growth status of vegetation. The performance of vegetation indicators is better, which may be due to the improvement in VER. For the comparison of data point clouds, the *white Birch* tree crown is closest to the actual measurement value, but the deviation of the trunk is relatively large, which may be due to the density of the branches. The monitoring effect of the growth of rough grass and *hydrangeas* can reach over 93%, which may be due to the better monitoring of shrub vegetation. In the process of ecological restoration, the growth of vegetation significantly improved, with the growth areas of rough grass and *hydrangeas* increasing by 5635 km^2^ and 7509 km^2^, respectively. Measures such as RFTFs can indeed promote vegetation growth. In vegetation coverage monitoring and analysis, the coverage area of Spartina alterniflora is closer to the true situation. However, there is a significant deviation between the growth and monitoring of *hydrangeas*, which may be due to the larger monitoring error caused by the wider growth range of *hydrangeas*. In the comparison of different regional models, region 3 had the highest number of single trees, and the vegetation coverage could reach 73.68%. The actual monitoring effect of region 3 was better in vegetation monitoring, which may be due to the fact that region 3 is mostly composed of tall trees. After implementing a series of policies, it was found that the vegetation restoration effect in the Daqing oilfield area had been increasing year by year with the increase in implementation time.

In summary, the use of MRS technology and LPC technology can effectively monitor the ecological restoration of regional vegetation. The analysis of the current ecological indicator restoration data proves that the committee has put forward good ecological restoration suggestions. This has great practical significance for the study of VER in this region.

## 5. Conclusions

MRS is more suitable for large-scale vegetation monitoring because it can quickly cover a large area and provide vegetation health and distribution data through spectral indices such as NDVI. LPC technology is more suitable for the detailed structural analysis of vegetation, with higher accuracy in measuring vegetation height, canopy density, and 3D structures. Therefore, MRS is more suitable for broader evaluations, while LPC excels in detailed structural evaluations. To achieve the best results, combining these two methods can leverage the advantages of each method and provide comprehensive analytical capabilities for vegetation health and restoration progress. In this study, MRS and LPC technologies were used to monitor the VER situation in the Daqing oilfield area, and the growth and coverage of different vegetation were monitored and analyzed to completely monitor the effect of ecological restoration in this area. The results demonstrate that vegetation parameters such as altitude and vegetation slope exhibit different positive and negative correlations with vegetation growth in different years. In 2023, the TWI of vegetation in the Daqing oilfield area was negatively correlated, and other parameters were positively correlated. The research method could achieve an estimated effect of over 90% in monitoring different vegetation cover conditions, and the monitoring effect of rhododendrons could reach 93.5%. The ecological restoration of different plants has improved, with the highest coverage area improvement of 7509 km^2^ achieved by *hydrangea* restoration. Research techniques could achieve good data results in comparison with several techniques and were closer to the actual coverage situation. In the process of point cloud monitoring, the area with the highest number of individual trees was area 3; the vegetation height could reach 13.64 m; and the vegetation width could reach a maximum of 7.68 m. The coverage of the area reached 73.68%. Therefore, MRS and LPC technologies can effectively monitor the situation of VER and have good monitoring effects. Although this study has achieved some results, there are still some shortcomings, such as only analyzing some plants. In future research, more plants will be analyzed. Secondly, this study only analyzes the Daqing oilfield region, and more regional situations should be discussed in later work.

## Figures and Tables

**Table 1:** Correlation between vegetation parameters at different time nodes.

r	FVC (2023)	FVC (2023–2010)	FVC (2010–2000)	FVC (2000–1990)
Elevation	0.163	0.131	−0.215	−0.244
Slope	0.347	0.245	0.143	0.066
Aspect	0.045	0.008	0.037	−0.042
TWI	−0.155	0.086	−0.068	−0.070

**Table 2:** Vegetation restoration in different regions.

Area Number	Amount of Vegetation in the Region	Vegetation Height (m)			Vegetation Width (m)			Vegetation Coverage (%)
		Min	Max	Average	Min	Max	Average	
1	215	0.34	10.35	8.64	1.23	11.57	5.54	72.24%
2	197	0.25	12.68	8.97	1.03	13.36	4.86	78.58%
3	268	0.36	13.64	7.68	0.89	16.68	6.87	73.68%
4	173	0.57	14.48	6.67	1.54	14.68	5.87	70.35%
5	234	0.48	16.84	9.87	0.76	13.75	2.42	74.88%

## Data Availability

The original contributions presented in the study are included in the article, further inquiries can be directed to the corresponding author.
